# Big Data in Cardiology: State-of-Art and Future Prospects

**DOI:** 10.3389/fcvm.2022.844296

**Published:** 2022-04-01

**Authors:** Haijiang Dai, Arwa Younis, Jude Dzevela Kong, Luca Puce, Georges Jabbour, Hong Yuan, Nicola Luigi Bragazzi

**Affiliations:** ^1^Department of Cardiology, The Third Xiangya Hospital, Central South University, Changsha, China; ^2^Laboratory for Industrial and Applied Mathematics (LIAM), Department of Mathematics and Statistics, York University, Toronto, ON, Canada; ^3^Clinical Cardiovascular Research Center, University of Rochester Medical Center, Rochester, New York, NY, United States; ^4^Department of Neuroscience, Rehabilitation, Ophthalmology, Genetics, Maternal and Child Health (DINOGMI), University of Genoa, Genoa, Italy; ^5^Physical Education Department, College of Education, Qatar University, Doha, Qatar; ^6^Postgraduate School of Public Health, Department of Health Sciences, University of Genoa, Genoa, Italy; ^7^Section of Musculoskeletal Disease, Leeds Institute of Molecular Medicine, NIHR Leeds Musculoskeletal Biomedical Research Unit, University of Leeds, Chapel Allerton Hospital, Leeds, United Kingdom

**Keywords:** Big Data, epidemiological registries, high-throughput technologies, wearable technologies, non-conventional data streams, cardiology

## Abstract

Cardiological disorders contribute to a significant portion of the global burden of disease. Cardiology can benefit from Big Data, which are generated and released by different sources and channels, like epidemiological surveys, national registries, electronic clinical records, claims-based databases (epidemiological Big Data), wet-lab, and next-generation sequencing (molecular Big Data), smartphones, smartwatches, and other mobile devices, sensors and wearable technologies, imaging techniques (computational Big Data), non-conventional data streams such as social networks, and web queries (digital Big Data), among others. Big Data is increasingly having a more and more relevant role, being highly ubiquitous and pervasive in contemporary society and paving the way for new, unprecedented perspectives in biomedicine, including cardiology. Big Data can be a real paradigm shift that revolutionizes cardiological practice and clinical research. However, some methodological issues should be properly addressed (like recording and association biases) and some ethical issues should be considered (such as privacy). Therefore, further research in the field is warranted.

## Cardiovascular Disease: Epidemiology and Global Burden of Disease

The global burden of disease (GBD) is the quantitative estimation of the health loss because of a disorder, risk factor, or injury. It is modeled and computed as the epidemiological, clinical, and societal burden generated by a given disease, in terms of its economic-financial and humanistic impact, if ineffectively managed and inadequately treated. Such a quantitative approach enables practitioners and scholars as well as all relevant stakeholders, including public and global health decisions- and policymakers, to compare the burden of different diseases, risk factors, or injuries, robustly and consistently over a temporal period and across various spatial settings and territories/nations. Moreover, these data can inform policies in a pure data-driven and evidence-based fashion, allowing prioritization and allocation of resources, especially in developing countries and in other resource-limited contexts (1). This approach enables to monitor the effects of a given policy or intervention and verifies if sufficient progress has been made toward the achievement of the Sustainable Development Goals (SDGs) set up by the United Nations (UN) General Assembly (2). In particular, SDG 3.4.1 has the ambitious goal of achieving a 30% reduction in premature mortality due to non-communicable diseases, including cardiovascular disease (CVD), by 2030 (2).

To track such a target, the GBD initiative as well as other similar taskforces and groups, like the Global Health Estimates (GHE) initiative led by the World Health Organization (WHO), have devised and implemented a set of validated and reliable indicators. These measures include the years of life lost (YLLs), the years lived with disability (YLDs), and the disability-adjusted life years (DALYs), which allow researchers to quantitatively evaluate life lost due to death (casualty or premature death) or disability, respectively, which hinder to live life at 100% health.

As previously mentioned, GBD- and GHE-related metrics are of paramount importance in providing stakeholders with data, especially in those settings where there is a dearth of data, or data are not properly updated and/or reliable, because they would be too much time- and resource-consuming to collect. CVD contributes to a significant portion of the GBD (3). CVD, especially stroke and ischemic heart disease (IHD), is the leading cause of mortality and disability. Prevalent CVD cases have nearly doubled from 271 million in 1990 to 523 million in 2019, globally. Similarly, the number of CVD deaths has increased from 12.1 million to 18.6 million, with DALYs and YLLs increasing as well. YLDs have doubled from 17.7 million to 34.4 million. Despite scholarly achievements and technological advancements, especially concerning the management of acute coronary artery disease, chronic ischaemic heart disease, and heart failure, CVD still imposes a dramatically high burden, which is increasing even in those settings in which it was previously decreasing (3, 4), pointing out the urgent need of implementing effective public health policies at a global and local level. This burden is still dramatically high for diseases, like atrial fibrillation, acute heart failure, or sudden cardiac death (3, 4).

In the present review paper, we will show how cardiology can benefit from the use of the so-called “Big Data”, especially in the efforts of counteracting and mitigating against the burden of CVD. In the next paragraphs, we will overview the changes cardiological research and practice have undergone in the last decades and we will make some examples of potential applications of Big Data in the cardiological arena, broken down according to their sources/channels (Tables 1–3), as well as their current major shortcomings and limitations (Table 4).

**Table 1:** Types of big data and their sources/channels in the field of cardiology.

**Type of big data**	**Sources/channels**
Epidemiological/clinical big data	Epidemiological survey
	Claims-based database (administrative database)
	Electronic health records (EHRs)/electronic medical records (EMRs)
	Large clinical registries (the “Society of Thoracic Surgeons (STS) National Database,” the “American Heart Association (AHA) Get With The Guidelines (GWTG) Database,” the “American College of Cardiology (ACC) National Cardiovascular Data Registry” (NCDR), the “Hospital Compare Database,” the “National Heart Lung and Blood Institute (NHLBI) Percutaneous Transluminal Coronary Angioplasty (PTCA) Registry,” the “STS/ACC Transcatheter Valve Therapy (TVT) database,” the “Hypertrophic Cardiomyopathy Registry,” and the “Cooperative Cardiovascular Project”)
Molecular big data	Microarray chips, next-generation DNA and RNA sequencing and whole-exome sequencing, chromatin-immunoprecipitation-coupled sequencing, and mass-spectrometry-based proteomics analysis
Big data generated by information and communication technologies (ICTs)	Smartphones, apps, and gamified mobile apps Smartwatches Sensors and wearable devices/technologies Imaging techniques (i.e., radiography, radiomics, and radiogenomics)
Computational/digital big data	“Non-conventional data streams”
	Web searches (Google Trends)
	Website page consultation (i.e., Wikipedia)

**Table 2:** Some select examples of big data-based registries/databases for cardiovascular disease.

**Country/territory**	**Database name/acronym**	**Major details**
Japan	Japanese Registry Of All cardiac and vascular Diseases-Diagnostic Procedure Combination (JROAD-DPC)	Governed by the Japanese Circulation Society (JCS), more than 700,000 health records' data as of 2012 from 610 certificated hospitals
	Japan Acute Myocardial Infarction Registry (JAMIR)	>20,000 patients
Korea	Prospective Cohort Registry for Heart Failure in Korea (KorAHF)	>5,000 patients
Denmark	Danish Cardiac Rehabilitation Database (DHRD)	Collecting data from all hospitals in Denmark
	Danish Heart Registry	Collecting data from five cardiology centers, eight cardiology satellite centers, four surgical centers, and a private hospital
Sweden	Swedish Primary Care Cardiovascular Database (SPCCD)	>70,000 patients
	SWEDEHEART	>2 million subjects
USA	National Cardiovascular Data Registry (NCDR)	Governed by the American College of Cardiology (ACC), it consists of 10 registries, eight inpatient/procedure-based and two outpatient-based from more than 2,400 hospitals and 8,500 providers with more than 60 million patient records

**Table 3:** Types of big data and examples of potential uses/applications in the field of cardiology.

**Type of big data**	**Examples of potential uses/applications**
Epidemiological/clinical big data	Epidemiological assessment (incidence, prevalence, co-morbidities, and mortality rates)
	Epidemiological nowcasting/forecasting for funding and resources allocation optimization
	Economic assessment (costs evaluation)
	Evaluation and comparison of different cardiological treatment and management options
	Identification of diagnostic and prognostic markers
	Evaluation and assessment of mid-term and long-term clinical outcomes
Molecular big data	Patient profiling and stratification
	Personalized/individualized cardiology
	Characterization of the effects and actions of drugs at the cellular and molecular levels
	Identification of potential druggable targets
Big data generated by information and communication technologies	Collection of patient-reported outcomes
	Customization and personalization of healthcare provision delivery
Computational/digital big data	Patient health-related literacy assessment
	Patient education and empowerment

**Table 4:** Major shortcomings and limitations of big data in current cardiological practice and clinical research.

**Type of big data**	**Limitation/issue**
Epidemiological/clinical big data	Discrepancies between registry-based studies and individual (single- or multi-center) investigations
	Discrepancies among database-based studies
	Privacy and bioethical issues
Molecular big data	Conflicting results among studies (depending on the type of tissue studied, the type of molecular technique used, etc.)
	“False discovery” of markers
Big data generated by information and communication technologies	Privacy and bioethical issues due to the pervasive and ubiquitous nature of the devices
Computational/digital big data	Lack of transparency concerning the algorithm

## Toward a New Way of Practicing Cardiology and Doing Cardiological Research

Healthcare provision delivery has changed dramatically in the last decades. New models and pathways of managing and treating diseases have emerged. A new biomedical approach termed “P4 medicine” (preventative, predictive, personalized, and participatory) has been introduced by Doctor Leroy Hood, a pioneering and inspiring figure in the arena of systems biology, pointing out the shift from a “one-size-fits-all” theoretical framework to one in which the individual signature of the disease matters (5–9).

Moreover, thanks to its latest scientific and technological improvements, medicine, including cardiology, is entering a new, unprecedented era, characterized by the production and release of an incredible amount of data, termed as Big Data. They are characterized by several key dimensions, including velocity (Big Data can be generated, processed, and analyzed in real-time), volume (referring to the wealth of data, the magnitude of which challenges classical storage, processing, and analytical capacities and infrastructures), variety (referring to the diversity of data sources, administrative, patient-reported, healthcare-generated, etc.), veracity (credibility, reliability, and accuracy of data), and value (raw data that, once processed, become smart, applicable, and actionable).

**Figure 1:**
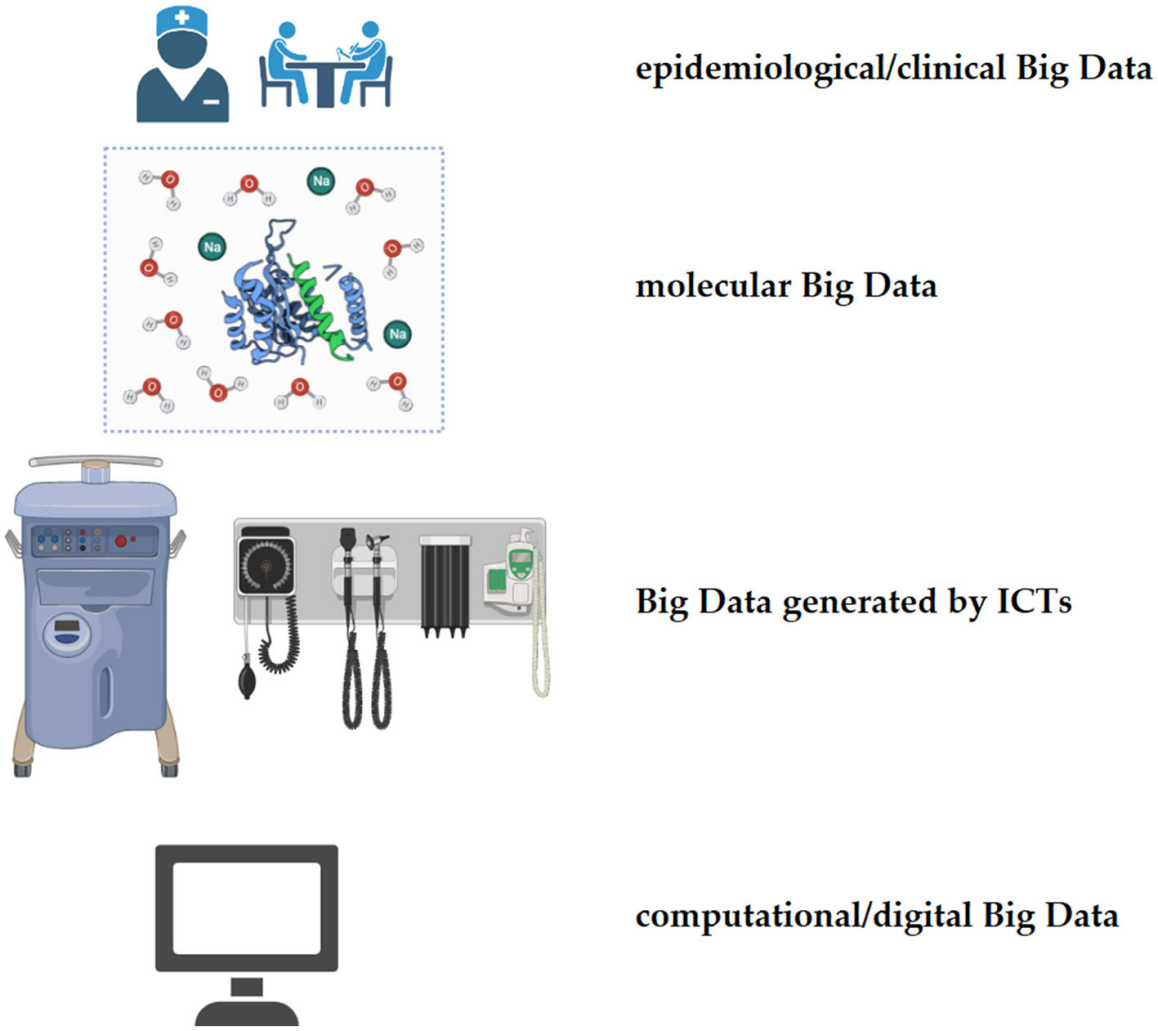
Types and sources/channels of big data.

**Figure 2:**
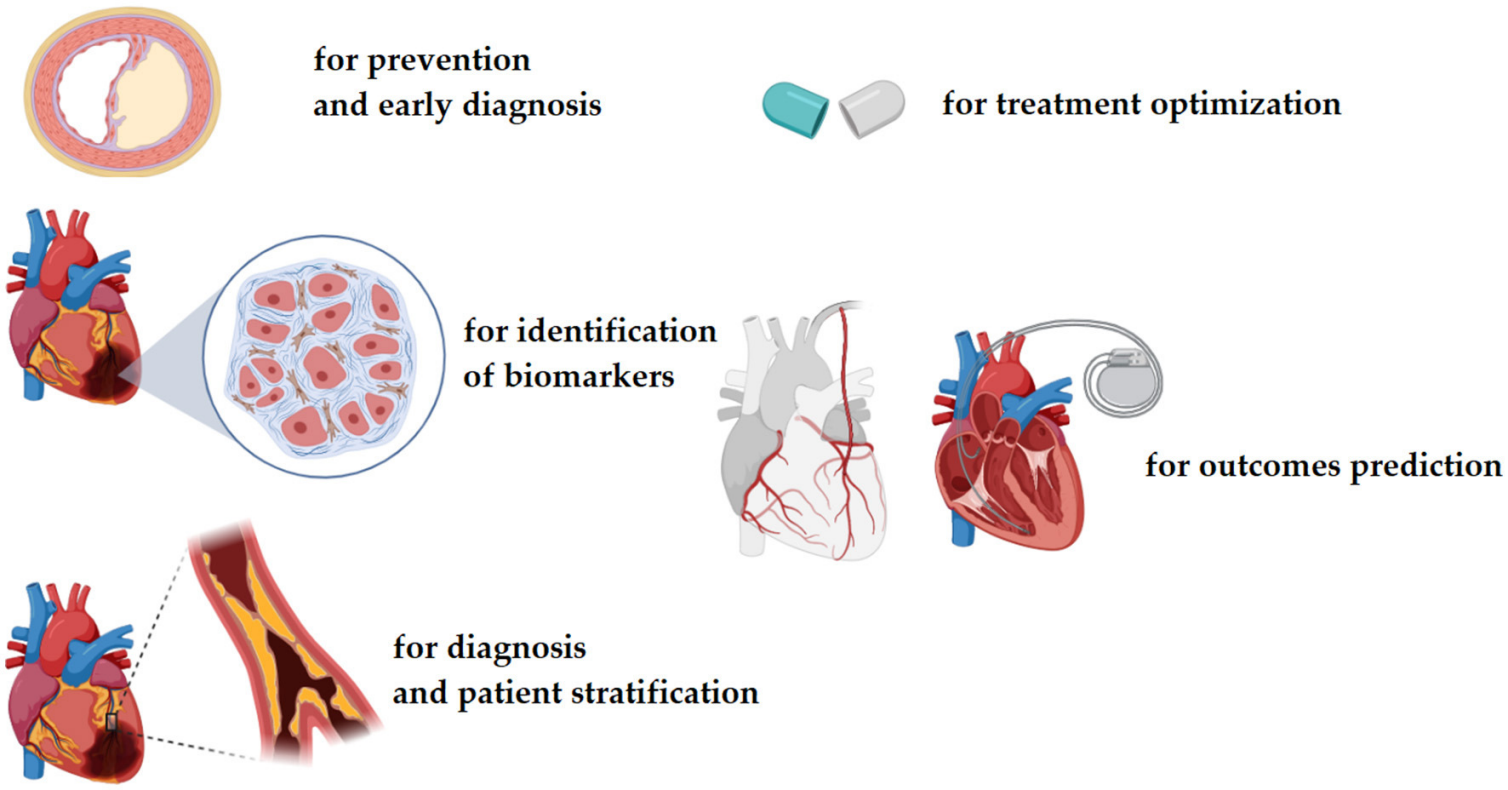
Potential applications of big data in the field of cardiology.

Different channels and sources can produce Big Data: from large-scale surveys, databases, repositories, and registries (epidemiological/clinical Big Data) to next-generation sequencing and high-throughput technologies (molecular Big Data) and computational approaches (infodemiological or digital Big Data). Big Data is deeply transforming clinical practices into disruptive ones and informing data-driven approaches (Figures 1, 2).

The “American College of Cardiology (ACC) Task Force on Health Policy Statements and Systems of Care” designed the 2017 Roadmap for Innovation in the cardiological arena, identifying three major pillars: namely, i) digital health, ii) Big Data, and iii) precision health (10, 11).

## Roles and Applications of Epidemiological/Clinical Big Data in Current Cardiological Research and Practice

Epidemiological/clinical Big Data can come from large-scale, often nationwide surveys. These data can inform public and global health policies as well as evidence-based medicine and, more specifically, cardiology.

Whilst randomized controlled clinical trials represent the gold standard for building a body of rigorous and clinically relevant evidence, they may not always reflect real-life patient populations, as such limiting the generalizability and external validity of their findings. Real-life or real-world evidence, collected during daily clinical practice, provides a complementary perspective to rigorously and strictly randomized controlled clinical trials (12, 13). In this respect, Big Data-based studies can add to well-designed “small data”-based investigations and randomized controlled clinical trials (13).

A major example of real-life or real-world data is TriNetX, which is the largest global research network providing real-world evidence. It contains tens of billions of clinical facts diagnosis, laboratory findings, treatment received, procedures performed, on more than 250 million patients worldwide, including subjects suffering from hypertensive disease, type 2 diabetes, or chronic kidney disease. Specifically, concerning the cardiological arena, this network has been exploited to shed light on the safety profile and cardiovascular outcomes of drugs (14, 15), the effectiveness of rehabilitation protocols (16, 17), and the cardiovascular implications of the still ongoing “Coronavirus Disease 2019” (COVID-19) pandemic (18, 19), among others.

To paraphrase what Doctor Lukas Kappenberger, pioneering father of the so-called “computational cardiology,” has stated in 2005, the science (i.e., randomized controlled clinical trials) tells scholars and practitioners what they can do, the guidelines and checklists implement what they should do, and clinical registries/databases tell them what they are doing and observing (20, 21).

Currently, there are lots of sources generating epidemiological Big Data, such as surveys, medical insurance data, vital registration data, cohort data, inpatient and outpatient data, among others (20).

These data can be retrospectively or prospectively collected: prospective clinical registries can be defined as large/very large datasets of observational data which have been collected prospectively and systematically and in a structured fashion, to reflect real-world clinical practices and outcomes of a given procedure (treatment, or surgical intervention) across large patient populations, including specific clinical/demographic (sub-)populations (20).

Furthermore, besides being complementary, randomized controlled clinical trials can be embedded within clinical registries (20): this enables to save time and resources and strengthens the generalizability of the findings (20). For instance, the “Coronary Artery Surgery Study (CASS) registry,” which is also one of the early examples of clinical registries, is a database embedded within a clinical trial (the CASS investigation) (22).

In the cardiological arena, there exist very large clinical databases and registries, whose origins can be dated back to the eighties (20). The most popular ones include societal registries, that is to say, databases endorsed, funded, and sponsored by scientific societies, like the “Society of Thoracic Surgeons (STS) National Database,” which collects clinical outcomes for patients undergoing cardiothoracic surgery (23), and the “American Heart Association (AHA) Get With The Guidelines (GWTG) Database,” which is based on a hospital-based initiative, led by the AHA and the American Stroke Association (ASA), collecting data from >2,000 hospitals, aimed at improving the quality of care of patients suffering from CVD, including heart failure, atrial fibrillation, and stroke (24). Another major societal database is the “ACC National Cardiovascular Data Registry” (NCDR), which is composed of 10 registries (eight of which are inpatient/procedure-based and the remaining two are outpatient-based), collecting data from >2,400 hospitals and 8,500 healthcare providers with >60 million patient records (25).

Other databases include the “Hospital Compare Database”, which collects data concerning the quality of care (overall star rating and other quality measures) from >4,000 Medicare-certified hospitals (26), and the “Cooperative Cardiovascular Project” (27, 28), which is one of the early examples of a clinical registry.

Some datasets and registries are devoted to specific cardiovascular medications or surgical procedures, like the “National Heart Lung and Blood Institute (NHLBI) Percutaneous Transluminal Coronary Angioplasty (PTCA) Registry”, collecting outcomes data for patients undergoing PTCA (29, 30), the “STS/ACC Transcatheter Valve Therapy (TVT) database”, which collects outcomes data for patients undergoing transcatheter valve replacement and repair procedures from >650 reporting sites (31), and the CathPCI registry from the NCDR, collecting outcomes data for patients undergoing diagnostic catheterization and/or percutaneous coronary intervention (PCI) procedures (32).

Some registries and databases are specifically devoted to particular CVD, like the “Hypertrophic Cardiomyopathy Registry” led by the University of Virginia (USA) and the University of Oxford (UK), aimed at identifying biomarkers of hypertrophic cardiomyopathy (33).

Epidemiological/clinical big data can be utilized for a variety of purposes and aims, including i) performing an epidemiological assessment of CVD (in terms of incidence, prevalence, and mortality rates), ii) quantifying and forecasting epidemiological trends, iii) investigating the determinants of CVD and related underlying co-morbidities, iv) identifying diagnostic and prognostic markers and signatures, v) devising risk score tools to better stratify CVD patients, vi) exploring the mid-term and long-term clinical outcomes of a given (pharmacological or surgical) procedure and its superiority over another one (the competitor), vii) verifying the implementation of recommendations and decision-making processes, setting benchmarks, and viii) computing the economic-financial costs of a given CVD (34, 35).

Big data can help uncover relationships between diseases and/or co-morbidities, in that they tend to co-cluster. The diseasome is the “human disease network”: a Big Data-based study of the diseasome can contribute to a better understanding of the so-called “system or network medicine” (36). Several CVDs, including heart failure, frequently coexist with various comorbidities. Meireles et al. (37) assessed the prognostic role and impact of several underlying comorbidities on the risk of developing acute heart failure. A set of 229 patients suffering from acute heart failure was compared vs. a set of 201 patients with chronic heart failure. The number of comorbidities was slightly higher in the acute heart failure patient group: these included metabolic impairments such as hyperuricemia, and obesity, other CVDs like atrial fibrillation, or peripheral artery disease as well as chronic kidney disease. Investigating the comorbidome could allow the implementation of “precision cardiology” by devising *ad hoc* multi-dimensional interventions targeting the specific patient sub-population.

There exist several risk tools, ranging from the Framingham score to the SCORE, the “Global Registry of Acute Coronary Events” (GRACE), the “Thrombolysis In Myocardial Infarction” (TIMI), the “Congestive Heart Failure, Hypertension, Age (≥75 years), Diabetes, Stroke, Vascular disease, Age (65 to 74 years), and Sex category” (CHA_2_DS_2_VASc), and the “Meta-Analysis Global Group in Chronic Heart Failure” (MAGGIC) risk-score, among others (38).

These risk calculators are fundamental components of the so-called “personalized cardiology,” in that they enable to stratify patient cohorts and provide the patient with the treatment they need the most. Examples of precision and personalized management include the customized assessment of the risk factor for a variety of cardiovascular diseases, such as atrial fibrillation, chronic myocardial ischemia, heart failure, and hypertension, given the individual biological makeup (genetic) and family history for cardiovascular disease. Also, pharmacological provisions, for instance, the usage of anticoagulants, can be tailored, in such a way to minimize the insurgence of potential side-effects (6–8).

There are, however, few published comparisons among the different risk scores, which remains a field open to further research and investigation (38).

Some emerging applications of Big Data-based databases are: i) addressing cardiovascular-related iniquities and disparities, also from a gender perspective, ii) performing post-marketing analysis of different cardiovascular treatments and medications (39).

Finally, Big Data is particularly helpful when the studied cardiological disease is rare, such as congenital CVD (40): pediatric cardiology is anticipated to benefit a lot from large datasets and the deployment of artificial intelligence (41).

Artificial intelligence is anticipated to fully leverage and harness Big Data-based databases, potentially overcoming the issue of “classical” and “conventional” statistical techniques, including propensity score analysis and multivariate regression modeling (42). Ahn et al. (43) developed CardioNet, a manually curated, standardized, and validated, comprehensive CVD-related database based on clinical information (either structured or unstructured) collected from 748,474 patients, that can be utilized for Artificial intelligence analyses and provide insights on the care of patients with CVD. Barbieri et al. (44) combined the classical survival analysis (Cox proportional hazard modeling) with a deep learning approach on a cohort of 2,164,872 New Zealanders aged 30–74 years. Predictors of CVD events were found to be tobacco use in women and chronic obstructive pulmonary disease (COPD) with acute lower respiratory infection in men, besides well-established risk factors like high blood pressure, chest pain, diabetes, and metabolic impairment.

On the other hand, despite the use of sophisticated statistical tools, as previously mentioned, there are still open issues that need to be addressed and solved. Big Data-based studies can offer a different point of view, but some conflicting findings of randomized controlled clinical studies and small, well-conducted investigations can be found.

Such discrepancies could be due to the unique nature of the database used in the study: each cardiological database significantly varies in the methods deployed to collect and capture data and the population(s) it specifically represents (13). Also, the format of the database (structured vs. unstructured) could impact data quality. For instance, Hernandez-Boussard et al. (13) mined a dataset inclusive of 10,840 clinical notes and found lower recall and precision rates (51.7 and 98.3%, respectively) in the case of structured electronic health records (HER), concerning unstructured EHR (95.5 and 95.3%, respectively), warranting the routine measurement of recall for each database/registry, before proceeding with data processing and analysis.

Summarizing, Big Data repositories, registries, and databases are increasingly common in the field of cardiological practice and clinical research: there are, however, significant considerable variations in socio-demographic characteristics, co-morbidities, and major complication rates between individual (single- or multi-center) and database-based studies, and even among registry-studies themselves (for example, clinical vs. administrative database). This should be accounted for when critically appraising cardiological research and in risk adjustment modeling (20).

In particular, administrative databases (20) can provide researchers and scholars, as well as practitioners and policy- and decision-makers with a lot of information concerning disease epidemiology, co-morbidities, disparities, and inequalities in access to healthcare and clinical outcomes. Furthermore, they can inform in a data-driven fashion the decision-making processes underlying cardiological pharmacological treatments or surgical procedures, in terms of pre-operative risk stratification parameters to significantly curb/minimize perioperative morbidity and mortality rates. On the other hand, administrative databases (20) may suffer from clerical inaccuracies, recording bias (due to the very nature of the database and secondary to economic-financial incentives underlying the collection, and maintenance of the dataset), temporal changes in nosology and nomenclature systems as well as in billing codes, and, finally, a dearth of several clinically relevant parameters, including cardiology-specific variables and outcomes.

A major issue seriously limiting the deployment of databases and registries is related to their inter-operability and sometimes inconsistent use of definitions. Moreover, not all databases meet regulatory standards (13) and are enough curated/validated. As such, data standardization and meta-data are urgently warranted (20).

Conversely, clinical studies, especially those relying on “Small Data,” even though well-designed and well-conducted, are generally statistically underpowered and are plagued by several biases, including participants sampling and selection bias, which hinders the generalizability of the findings, with samples being not representative of the entire population. It is also difficult to stratify according to a given cardiological pharmacological treatment or surgical procedure if the sample is particularly heterogeneous and the sample size does not allow to make sufficiently statistically robust and reliable calculations. Confidence and certainty can increase with “Big Data,” paralleling, however, the growth of complexity and associated computational costs (45, 46). Also, Big Data-based databases can be affected by biases, as previously mentioned, such as recording or association biases and other statistical artifacts, like “reverse epidemiology” or “reverse causality” (47). For instance, some database-based studies have found that body mass index (BMI), lipid profile, and blood pressure, which usually predict a poor clinical outcome in the general population, become inverse prognostic predictors in chronic heart failure patients. Greater survival has been, indeed, linked to overweight and obesity, hypercholesterolemia, and high values of blood pressure, which is rather counter-intuitive. Several hypotheses have been formulated, including the presence of the “malnutrition-inflammation complex syndrome” or “malnutrition–inflammation–cachexia syndrome”. However, some scholars think that it is more likely (and biologically/clinically plausible) that these findings are statistical artifacts.

## Roles and Applications of Molecular Big Data in Current Cardiological Practice and Research

Wet-lab and high-throughput technologies, including microarray chips, next-generation DNA and RNA sequencing and whole-exome sequencing, chromatin-immunoprecipitation-coupled sequencing, chromatin interaction analysis by paired-end tag sequencing (ChIA-PET), chromatin conformation capture with sequencing, assay for transposase-accessible chromatin with high-throughput sequencing (ATAC-Seq), and mass-spectrometry-based proteomics analysis can generate a wealth of molecular big data, paving the way for a personalized/individualized rather than “one-size-fits-it-all” cardiology (48, 49).

Molecular big data can elucidate the mechanisms underlying the etiopathogenesis of a given heart disease and identify new potential druggable targets for the development of *ad hoc* pharmacological therapies. Personalized cardiology can benefit from genome-wide association and post-genomics studies (50, 51), aimed at the identification of new cardiogenic transcription factors, genotypic and phenotypic validations of potential transcriptional regulators, and molecular/cellular mechanisms.

CardioGenBase (50) is a literature-based, comprehensive online resource tool, which extensively collects gene-disease associations (over 1,500) for major CVD, including cerebrovascular disease, ischemic heart disease, coronary artery disease (CAD), inflammatory heart disease, rheumatic heart disease, and hypertensive heart disease, among others.

Vakili et al. (51) made efforts to combine all the OMICS-based specialties within a highly integrated, coherent, multi-OMICS approach termed as “panomics,” to shed light on the multi-factorial pathogenesis of CVD. The authors systematically mined the literature and were able to find 104 CVD-related OMICS-based databases, 72 of which provided genomics/post-genomics and clinical measurements. Of these datasets, 59 and 65 databases were transcriptomic, epigenomic/methylomic, 41 proteomic, 42 metabolomic, and 22 microbiomic.

Combing the scholarly literature, clinical and OMICS-based information, and exploiting the “diseasome” approach, Sarajlić et al. (52) assessed the structure of the human protein-protein interaction (PPI) network to discover new CVD-related genes, that could be potential druggable targets. The authors found that these new genes were involved in intracellular signaling cascades, signaling transducing activity, enzyme binding, and intracellular receptor-mediated signaling pathways.

Moreover, the unique and unprecedented convergence between different disciplines, such as nano-(bio-) engineering, three-dimensional (3D) printing and computational simulation, molecular and mathematical modeling, and advanced and sophisticated biostatistical techniques and Artificial Intelligence (Data Mining, Machine, and Deep Learning), are shaping new paths and opportunities in the field of cardiological practice and clinical research, enriching it, making it more multi- and inter-disciplinary and complex, and more able to address the biomedical challenges. Similarly, Dr. Elias Zerhouni (53–55), who has served as Director of the National Institutes of Health (NIH) from 2002 to 2008, has indicated such a unique convergence as the future roadmap in the field of scholarly research, including the cardiological arena.

3D printing is being increasingly utilized in biomedicine, and, in particular, in cardiology. Generally, mainly rigid anatomic models are produced, but the incorporation of dynamic functionality is expected to dramatically advance preoperative cardiovascular surgical planning as well as hemodynamics (56). 3D models can shed light on different CVD-related pathophysiological conditions, thus complementing information obtained using classical imaging.

Moreover, molecular Big Data, alone or combined/integrated with epidemiological Big Data, can capture the landscape of several cardiological diseases and events, either idiopathic or congenital, including dilated cardiomyopathy and heart failure (57, 58), among others.

## Roles and Applications of Big Data Generated by Imaging Techniques and Wearable Technologies/Smart Sensors in Current Cardiological Practice and Research

Latest technological achievements in the field of mobile health (mHealth) and ubiquitous health (uHealth), with smartphones, smart devices, smartwatches, and other wearable sensors (59) are revolutionizing the field of cardiology, directly involving, and engaging the patient, improving their therapeutical adherence and compliance, and also enabling remote patient monitoring.

Wearable sensors of different types (bioelectric, mechano-electric, optoelectronic, and ultrasonic wearable devices) enable collecting cardiovascular vital signs (such as blood pressure, heart rate and heart rhythm, blood oxygen saturation, and blood glucose, as well as brain waves, air quality, exposure to radiations, and other metrics) continuously, allowing early intervention (60).

Gandhi et al. (61) conducted a systematic review of the literature, investigating the effectiveness of mHealth Interventions for the secondary prevention of CVD. The authors pooled 27 studies together, totaling 5,165 patients. mHealth was found to increase therapeutic adherence (with an odds ratio, OR, of 4.51) as well as overall compliance, either pharmacologic or non-pharmacologic (with an OR of 3.86). Different targets were more likely to be met: namely, blood pressure (OR 2.80), exercise and physical activity with reduced sedentary time and sitting (OR 2.55), but not smoking cessation (OR 1.42), and lipid profile (OR 1.16). However, the mHealth group did not differ from the standard-of-care group in terms of hospitalizations and hospital readmissions (OR 0.93). Few studies showed a statistically significant reduction in angina (OR 0.23) and transient ischemic attack/stroke recurrence in cerebrovascular disease patients (OR 0.18). The cardiovascular mortality rate was computed to be lower, even though not achieving the significance threshold (OR 0.19). Similar results could be replicated in a more updated systematic review and meta-analysis conducted by Akinosun et al. (62) and in the systematic review of the literature by Spaulding et al. (63).

Wali et al. (64) showed that mHealth interventions can be particularly useful in reaching vulnerable and underserved communities, including aboriginal and indigenous individuals or subjects residing in low- and middle-income countries. Usually, these individuals are excluded or are under-represented in clinical trials.

Gamification and gamified mobile applications (apps) represent another interesting and promising ramification of the digital health arena. Davis et al. (65) have performed a systematic literature review, synthesizing seven studies, totaling 657 patients. The authors found that gamification resulted in improved adoption of healthier lifestyles and behaviors (for instance, in terms of the practice of exercise and physical activity), better biochemical profile, enhanced mood, and motivation. Interestingly, also CVD-related health literacy and knowledge improved in a significant way, even though some parameters, such as blood pressure, body mass index, self-management, and therapeutical compliance, were comparable with standard-of-care.

To summarize, mHealth and digital health-based interventions, including telemonitoring (telecardiology) or text messaging, can be customized, meeting the needs of “personalized cardiology,” also becoming culturally sensitive and targeting specific populations, which are disproportionately affected by non-communicable diseases, including CVD.

Concerning smart devices, such as smartwatches and smartphones, Prasitlumkum et al. (66) have conducted a systemic review and meta-analysis to quantitatively evaluate the accuracy of utilizing wearable devices for screening, detecting, and properly diagnosing atrial fibrillation. The authors were able to compute excellent areas under the summary receiver operating characteristic (SROC) curves at 0.96 and 0.94, for smartphones and smartwatches, respectively. Sensitivity and specificity were in the range of 94–96 and 93–94% for the two kinds of smart/wearable devices, respectively: they proved to be as diagnostically accurate and reliable as gold standards, like photoplethysmography and single-lead electrocardiography.

Signals and data generated by imaging techniques, like electrocardiography, computed tomography, or magnetic resonance imaging, can be further processed, analyzed, and refined using artificial intelligence (67, 68). For instance, MOCOnet (69) is a next-generation convolutional neural network that can significantly enhance and improve quantitative cardiovascular magnetic resonance T1 mapping, making it more robust, reliable, clinically meaningful, less prone to motion artifacts, and in a time-efficient manner. MOCOnet, being purely data-driven, outperforms currently available methods for motion correction, which are model-driven.

Finally, radiomics and radiogenomics are highly innovative translational fields of research aimed at mining, retrieving, merging, processing, analyzing, and extracting clinically meaningful patterns and interpretations from large-scale, high-dimensional datasets generated by clinical imaging techniques and tools (70), including cardiac computed tomography angiography and cardiac magnetic resonance. Latest advancements concerning more and more sophisticated protocols enable the integration of imaging features and molecular profiling to identify relevant and clinically meaningful biomarkers and signatures (such as atherosclerotic lesions, coronary plaques, and myocardial structural abnormalities) related to diagnosis, prognosis, and response to treatment. Supervised and unsupervised artificial intelligence, including deep and machine learning, can further combine and aggregate data and assist the development of risk models and tools that can facilitate clinical diagnostic and prognostic procedures.

In the field of cardiological research, radiomics, and radiogenomics can be utilized for the characterization, profiling/phenotyping, and risk stratification of coronary heart disease (CHD), hypertrophic cardiomyopathy, ischemic heart disease, and cerebrovascular disease (70–73), among others. However, also given its recency, still too much has to be explored in this field. On the other hand, it can be anticipated that radiomics, radiogenomics, and other Big Data generated by wearable/smart devices and sensors will profoundly impact both cardiological practice and research.

## Roles and Applications of Infodemiological Big Data in Current Cardiology Research

Infodemiology (a *portmanteau* of “information” and “epidemiology”) and infoveillance (a combination of the words “information” and “surveillance”) represent a highly innovative discipline, at the intersection of computer, data, and behavioral science, aimed at shedding light on the determinants of computational and digital activities (such as web queries, use of social media, posting on social networks, and production/consumption of online material) (74, 75).

Researchers in the field of infodemiology and infoveillance make use of resources that enable to assess information demand and consumption, such as Google Trends, which is an open-source tool that enables to track and monitor web searches conducted using the Google search engine.

Infodemiology and infoveillance enable to track the effectiveness of awareness campaigns, such as the “Go Red for Women” (76), which is a social initiative aimed at improving and enhancing CVD- and stroke-related literacy among women. Suero-Abreu et al. (77) investigated the impact of “Go Red for Women” on health information-seeking behavior, utilizing Google Trends. Authors found increased search volumes related to the awareness campaign and various CVD-related terms over 15 years. However, stroke-related digital searches were not found to be increased over the study period.

Dzaye et al. (78) have exploited infodemiology and infoveillance techniques to assess public interest toward CVD and related comorbidities during the “Coronavirus Disease 2019” (COVID-19) pandemic. According to some studies, attention to CVD would have decreased, despite the negative relationship between CVD and infection. Patients suffering from CVD or with risk factors for CVD have been consistently reported to exhibit worse outcomes than their CVD-free counterparts. Authors found that digital interest in terms like exercise or physical activity and cigarettes had increased (by 18%) and decreased (by 52.5%), respectively. Noteworthy, interest in terms like statin, lipid profile, low-density lipoprotein (LDL), and hemoglobin A1C, had significantly increased at well, after a previous decline over time.

On the other hand, according to a research study by the same group (79), the first months of the COVID-19 pandemic were paralleled by a decrease in search interest for myocardial infarction and acute coronary syndrome (ACS), potentially explaining the excess cardiovascular mortality despite a marked reduction in hospitalization for ACS.

To summarize, search engines and other non-conventional data streams appear to be valuable and promising tools that can provide insights on health information-seeking behaviors and evaluate the effectiveness of social campaigns and other interventions.

The quality of cardiology-related websites and, more in general, online material is highly heterogenous and variable both in terms of content and information provided. For instance, Azer et al. (80) assessed the quality, accuracy, and readability of Wikipedia pages concerning CVD. About 83% of Wikipedia pages were deemed of moderate quality, with 8.5% being of good and poor quality, respectively. Despite clinical presentation and etiopathogenesis of CVD being treated and discussed, several sections, including the pathophysiology, signs and symptoms, diagnosis, and management, were not always accurate and adequately scholarly referenced. Several entries exhibited errors and omissions. The readability was at the level of collegiate subjects.

CVD patients use the internet as a low-cost and easily available source of personal healthcare information, to learn more about their condition/disorder, as well as about potential treatment options and CVD physicians and surgeons (81). According to a recent survey by Jones et al. (81), 74.3% of the interviewees surfed the internet, with 63% utilizing it daily. In the case the patient could not directly access the web, a family member was willing to do so on their behalf. The authors concluded that most patients (~85%) utilized the internet, being particularly interested in local information.

Practitioners and residents in the field of cardiology should be aware of these findings in that the web is often consulted by patients with CVD. Locally delivered Web-based information service is particularly requested and appreciated by CVD patients. The web can be used to deliver high-quality, educational material and empower the patient, by enhancing their literacy, collecting patient-generated/reported outcomes (PROMs), and health-related behaviors and attitudes, devising *ad hoc* social campaigns and monitoring their impact on health-related digital seeking behaviors.

## “Participatory Cardiology”: Integrating Basic and Translational Cardiology and Citizen Science

Big Data can also contribute to an emerging super-specialization within the field of cardiology: the so-called “participatory cardiology”, in such a way promoting public participation in the field of cardiological practice and clinical research, creating “global collaborative social networks”, and integrating basic and translational cardiology and citizen science (82, 83). This is of paramount importance especially in low- and middle-income countries and would help curb/reduce health disparities and iniquities.

A systematic review conducted by Wali et al. (64) has shown that establishing collaborative partnerships and relationships with community members – especially those from underserved and vulnerable populations – would significantly improve and enhance the effectiveness of the cardiological intervention by ensuring it was devised and implemented within the appropriate context.

Participatory cardiology, as a branch of participatory medicine, gives a new value and importance to the patient, who is the “real teacher,” quoting a famous statement of the Canadian physician and cardiologist Sir William Osler (1849–1919) enunciated in 1903. Latest scientific and technological advancements and current trends in clinical practice and research, especially in the cardiological arena, have gradually shifted the practitioners' attention and interest toward patient's “subjective” outcomes (satisfaction, pain, quality of life, etc.), besides “objective” clinical outcomes (healthcare resources uptake and consumption, healthcare processes and provisions delivery, morbidity and mortality rates).

However, for most cardiologists and cardiological surgeons, the world of PROMs represents a still “unchartered health care environment” (84), the navigation of which, by incorporating “mission, values and culture” (85, 86), can advance cardiological practice and research. There are several gaps in the implementation and full incorporation of PROMs within the daily routine cardiological practice. According to the “International Consortium for Health Outcomes Measurement” (ICHOM), while there exist several national, international, and trial registries for heart failure, very few of them can be considered as patient-centered and standardized guidelines and checklists guiding the process of properly, effectively, and meaningfully using PROMs are lacking. To fill in this gap, the ICHOM has developed a 17-item dataset, which consists of several domains (functional-, psycho-social-, burden of care-, and survival-related outcomes). This set, which also includes PROMs besides clinical/objective measurements, and administrative data, enables to compare consistently heart failure management and treatment across several healthcare providers and various regions, globally (87).

## Limitations and Shortcomings of Big Data in the Field of Cardiology

Table 4 overviews the major limitations and shortcomings of Big Data in the field of cardiology based on the type of source/channel that generates them. Basically, these pitfalls are of a two-fold nature: legal/bioethical (in terms of legal requirements and restrictions, legislation, privacy, and data sharing policies) and methodological.

Epidemiological/clinical Big Data can be affected by inconsistencies according to the type of study and its design (registry-based *vs*. individual – single or multi-center – investigations). Also, database-based studies may give rise to contrasting findings based on the reason and scope data were collected (clinical, administrative, or financial purposes). Optimizing databases and ensuring inter-operability could overcome these issues. Moreover, datasets can also be publicly uploaded and shared, enabling other scholars and researchers to replicate findings. However, there exist some privacy and bioethical issues. Data de-identification or anonymization or pseudonymization or masking can ensure re-use of potentially sensitive, personal, and legally restricted data, preserving scalability and performance, also if this technique could be challenging and not trivial to implement (88).

Molecular big data require extensive processing of data, which can be quite expensive, time- and resource-consuming. Moreover, the results of the various studies have to be reconciled, depending on the type of tissue/cell studies, the molecular technique applied, etc. This can lead to a “false discovery” of biomarkers. Recently, meta-analyses of molecular big data pooling together various samples have enabled to increase the statistical power and, thus, the reliability and trustworthiness of the discovery. Ensuring reproducibility and clinical meaningfulness of results should be a research priority (89).

Big data generated by information and communication technologies can be affected by privacy and bioethical issues due to the pervasive and ubiquitous nature of the devices.

Finally, concerning computational/digital big data, there are some issues affecting their usage, like the lack of transparency related to the algorithm deployed to retrieve, collect, process, and store data.

## Conclusions and Future Prospects

Big Data is increasingly having a more and more relevant role, being highly ubiquitous and pervasive in contemporary society, permeating it and paving the way for new, unprecedented perspectives in biomedicine, including cardiology. Big Data can be a real paradigm shift that revolutionizes cardiological practice and clinical research. However, some methodological issues should be properly addressed, and some ethical issues should be considered. Therefore, further research in the field is urgently warranted.

## Author Contributions

HD and NB conceived and drafted the paper. All other authors critically revised it. All authors contributed to the article and approved the submitted version.

## Conflict of Interest

The authors declare that the research was conducted in the absence of any commercial or financial relationships that could be construed as a potential conflict of interest.

## Publisher's Note

All claims expressed in this article are solely those of the authors and do not necessarily represent those of their affiliated organizations, or those of the publisher, the editors and the reviewers. Any product that may be evaluated in this article, or claim that may be made by its manufacturer, is not guaranteed or endorsed by the publisher.
